# Cholesterol derivatives make large part of the lipids from epidermal molts of the desert-adapted Gila monster lizard (*Heloderma suspectum*)

**DOI:** 10.1038/s41598-020-74231-5

**Published:** 2020-10-14

**Authors:** Cristian Torri, Giuseppe Falini, Devis Montroni, Simona Fermani, Roberta Teta, Alfonso Mangoni, Lorenzo Alibardi

**Affiliations:** 1 Department of Chemistry “Giacomo Ciamician”, University of Bologna Campus of Ravenna, via S. Alberto 163, Ravenna, Italy; 2 Department of Chemistry “Giacomo Ciamician”, University of Bologna, via Selmi 2, 40126 Bologna, Italy; 3 Department of Pharmacy, University of Napoli Federico II, via Montesano 49, 80131 Napoli, Italy; 4 Comparative Histolab Padova, Padua, Italy

**Keywords:** Structural biology, Zoology, Chemistry, Materials chemistry

## Abstract

In order to understand the cutaneous water loss in the desert-adapted and venomous lizard *Heloderma suspectum*, the microscopic structure and lipid composition of epidermal molts have been examined using microscopic, spectroscopic and chemical analysis techniques. The molt is formed by a variably thick, superficial beta-layer, an extensive mesos-region and few alpha-cells in its lowermost layers. The beta-layer contains most corneous beta proteins while the mesos-region is much richer in lipids. The proteins in the mesos-region are more unstructured than those located in the beta-layer. Most interestingly, among other lipids, high contents of cholesteryl-β-glucoside and cholesteryl sulfate were detected, molecules absent or present in traces in other species of squamates. These cholesterol derivatives may be involved in the stabilization and compaction of the mesos-region, but present a limited permeability to water movements. The modest resistance to cutaneous water-loss of this species is compensated by adopting other physiological strategies to limit thermal damage and water transpiration as previous eco-physiological studies have indicated. The increase of steroid derivatives may also be implicated in the heat shock response, influencing the relative behavior in this desert-adapted lizard.

## Introduction

The complex epidermis of snakes and lizards comprises different corneous layers that play specific roles for the integrity of the integument, contributing to the adaptation of these reptiles to their specific environment^1–6^. While the external beta-layer mechanically protects the skin, the underlying corneous layers, indicated as mesos- and alpha-layers, form the barrier against water-loss. The alpha-layer also allows the inter-scale stretching during movement and the plasticity of the scales during growth and shedding. Previous studies have concluded that most of the barrier against water-loss and its control resides in the mesos-region of the epidermis, whose cells contain specific lamellate granules, indicated as mesos-granules and lipids^7–10^.

The results of an extensive research activity indicate that diverse lipids are responsible for the regulation of water and ions permeability in the epidermis of snakes. Among polar and neutral lipids, also sterol esters, acylglucosyl-ceramides, glucosylsterols and acylglucosylsterols have been reported^8, 11^. Lipids extracted from snake molts include mainly triacylglycerols and a lower amount of sterols and acyl-sterols. Ceramides, glycerol and un-identified choline compounds have also been reported as minor components. Studies on the permeability of the epidermis in snakes with different ecological adaptations living at different environmental temperature and humidity were performed. They have shown that lipids present in mesos- and alpha-layers have a crystalline packing. These lipids change crystalline packing when the temperature of the environment increases. This structural re-organization, associated with the increase of temperature, reduces the water movement across the epidermis of snakes^12, 13^.

In order to extend the information on the process of water transpiration on xeric-adapted reptiles, we here examined the composition and structure of the epidermis in the Gila monster, *Heloderma suspectum.* In fact, this venomous lizard presents peculiar physiological and morphological adaptations in the dry conditions of the Sonoran Desert of the American Southwest^14–17^. These studies have indicated that the Gila monster shows total Evaporative Water Loss (EWL) higher to that of lizards adapted to non-arid environments, and instead utilizes other physiological and behavioral mechanisms for being active in arid conditions despite the loss of water^14, 16^. Also the morphology of the skin shows some adaptations to the arid environment and previous microscopic analysis of the epidermis in this species has shown the presence of a thick mesos-region that comprises over 50 thin mesos-cells in some regions of the scales and in the hinge region, representing the thickest mesos-layer ever recorded in the epidermis of reptiles^18^. The presence of such a thick mesos-region, unique among squamates, may indicate that lipids of different types are abundant in the epidermis of this species, probably in relation to water conservation. Other complex lipophilic molecules (carothenoids and pteridins), present in the dermal xanto-melanosomes, protect the skin from sun-light radiation more than from water-loss, another adaptation of this lizard to desert conditions^19^. Here we have extended the study of the structure and chemical composition, in particular of the lipid fraction, present in the mature shed epidermis (molts) of *H. suspectum*. The study aims to characterize the specific lipids that most likely are responsible for the peculiar desert adaptation of this unique species of lizard^14, 17^. The present study is part of a large-scale survey on the structure and composition of the epidermal molts of snakes and lizards and it can have implications on the control of water permeability through the epidermis of reptiles adapted to different ecological lifestyles.

## Results

### Microscopic structure of the epidermis and molts

**Figure 1:**
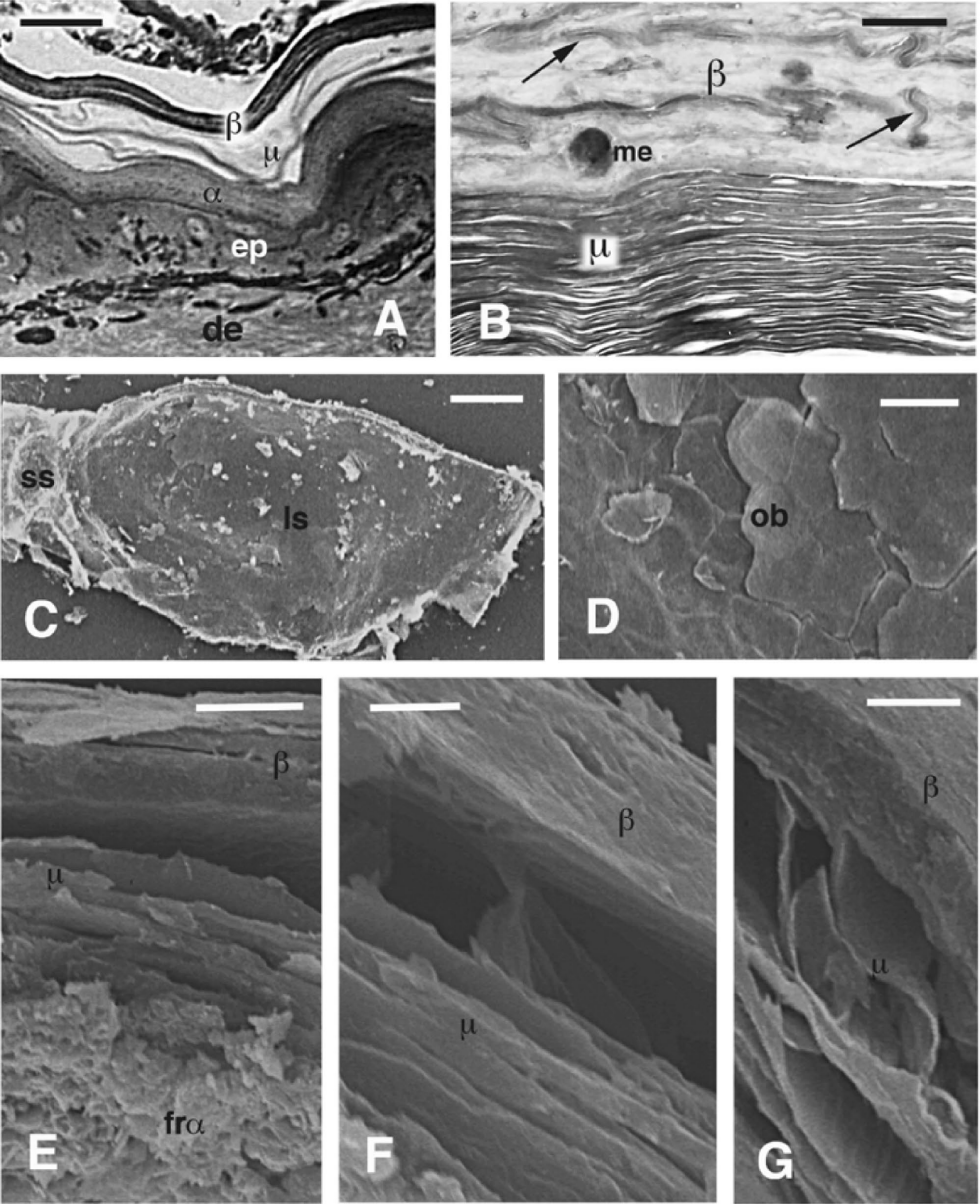
Histology (**A**) and TEM (**B**) images of the epidermis and SEM features of the molt (C-G) in *H. suspectum*. (**A**) epidermal layers. Bar, 10 μm. (**B**) TEM detail on the numerous thin mesos-cells present beneath the beta-layer (arrows indicate few desmosome remnants). Bar, 0.5 μm. (**C**) whole view of the smooth surface of scales (molt). Bar, 300 μm. (**D**) detail on the tile-like surface of a scale. Bar, 250 μm. (**E**) sequential layers from the external one inward. Bar, 10 μm. (**F**) detail on the beta and mesos-layer. Bar, 5 μm. (**G**) detail on mesos cells showing thin scale-like shape. Bar, 10 μm. *α* alpha-layer, *β* beta-layer, *de* dermis, *ep* epidermis, *frα* fragmented alpha-layer, *μ* mesos-layer, *me* melanosome, *ls* large scale, *ss* small scale.

Under light microscopy the epidermis of *H. suspectum* consists of a beta-layer with a greater thickness in the center of scales that becomes thinner toward the hinge region, while a thick mesos-region is present underneath (Fig. 1A). The ultrastructural study conducted by TEM shows an external, compact and electron-pale beta-layer, followed by a mesos-layer where numerous thin mesos cells, below 0.5 μm in thickness, form a piled and lamellar structure (Fig. 1B; see details in Alibardi and DeNardo, 2013)^18^.

The SEM observations show that the scale surface is relatively smooth (Fig. 1C), with outermost epidermal (Oberhautchen) cells with a tile-like patterned organization and whose surface is mostly smooth (Fig. 1D). The SEM observations through the thickness of the molts reveal that while the beta-layer is compact, the underlying mesos-layer shows detached and flat cells with broad intercellular spaces (Fig. 1E,F). SEM observations also clearly show the flat, scale-like 3D shape of mesos-cells (Fig. 1G). The variably extensive intercellular spaces are likely derived from mechanical stress associated with the preparation of the molt samples.

### Corneous material and lipid distribution in the molt

**Figure 2:**
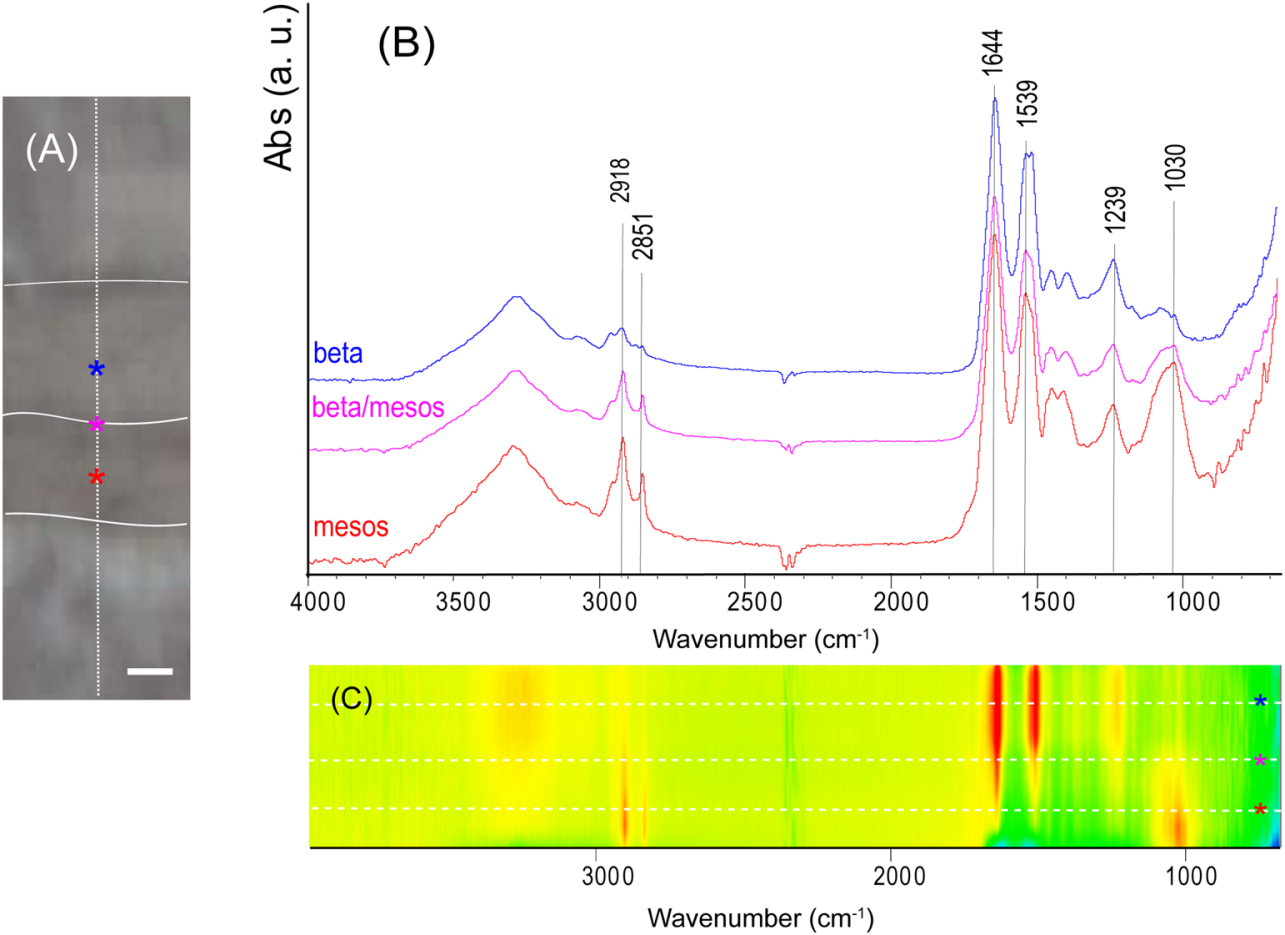
Lipids and keratins distribution in epidermal molts of *H. suspectum*. (**A**) Camera image of a cross section of the molt. The white dotted line indicates the scanned line during the ATR FTIR data collection. The upper and lower curved white lines delimitate the border between the molt and the KBr matrix. The middle white line separates the beta-layer (up) from the mesos-layer (bottom). The asterisks indicate the point where the ATR-FTIR spectra shown in (**B**) were acquired. The pseudo-color map (**C**) reports all the spectra collected along the cross-section. The dashed lines show where the spectra showed in (**B**) where collected along the cross-section. The intensity of the peak is scaled between the blue (minimum) and the red (maximum) colors. Bar in (**A**) 1 μm.

The *H. suspectum* molt shows a compositional layered structure, evident in the fluorescent images (Fig. S1) and from the attenuated total reflection Fourier Transform Infrared Spectroscopy (ATR-FTIR) spectra, analyzed in cross-section (Fig. 2). All the ATR-FTIR spectra collected along the cross-section (Fig. 2A) show the same absorption bands (Table S1), typical of corneous proteins and lipids, but with different relative intensities. This can be easily observed in the false color map shown in Fig. 2C, which reports the intensity of the bands along the cross section, normalized on the most intense band present for each spectrum. The band corresponding to the lipid absorption region, around 2920 cm^−1^, is more intense in the region of the molt that corresponds to the mesos-region than in the beta-layer. The region of transition from beta-layer to mesos- layer also shows a strong absorption in the region around 2920 cm^−1^ (Fig. 2A). The amide I absorption band is always centered at 1644 cm^−1^, but it shows a profile for the mesos-layer different for those from the beta-layer and the transition beta/meso-layer, which are almost superimposable. The fit of this band is performed using three contributions: (1) a band centered around 1644–1650 cm^−1^ associated with α-helices; (2) a band centered around 1620–1624 cm^−1^ associated with β-sheets; and (3) a band centered around 1674–1678 cm^−1^ associated with turns, loops and random coils^20^. The analyses of α-helix, β-sheet and turns, loops, random coils components (Fig. S3, Table S2) of this band reveals that in the mesos-layer the relative content of the disordered structure increases by about 10% while there is a decrease of the α-helix structure with respect to the beta- and the transition beta/mesos-layer. The relative amount of β-sheet structures does not change along the cross-section of the molt. The amide II band shows a profile in the beta-layer that is different from the profiles detected in the other two internal layers. This is due to the higher intensity of the component at about 1512 cm^−1^ (see Table S2). The analysis of the ATR-FTIR profile also reveals that the relative integrate intensity of the bands in the region 2800–3000 cm^−1^ in the mesos-layer is about 52% higher than that in the beta- and beta/mesos-layer.

### Chemical composition of the lipidic layer

**Figure 3:**
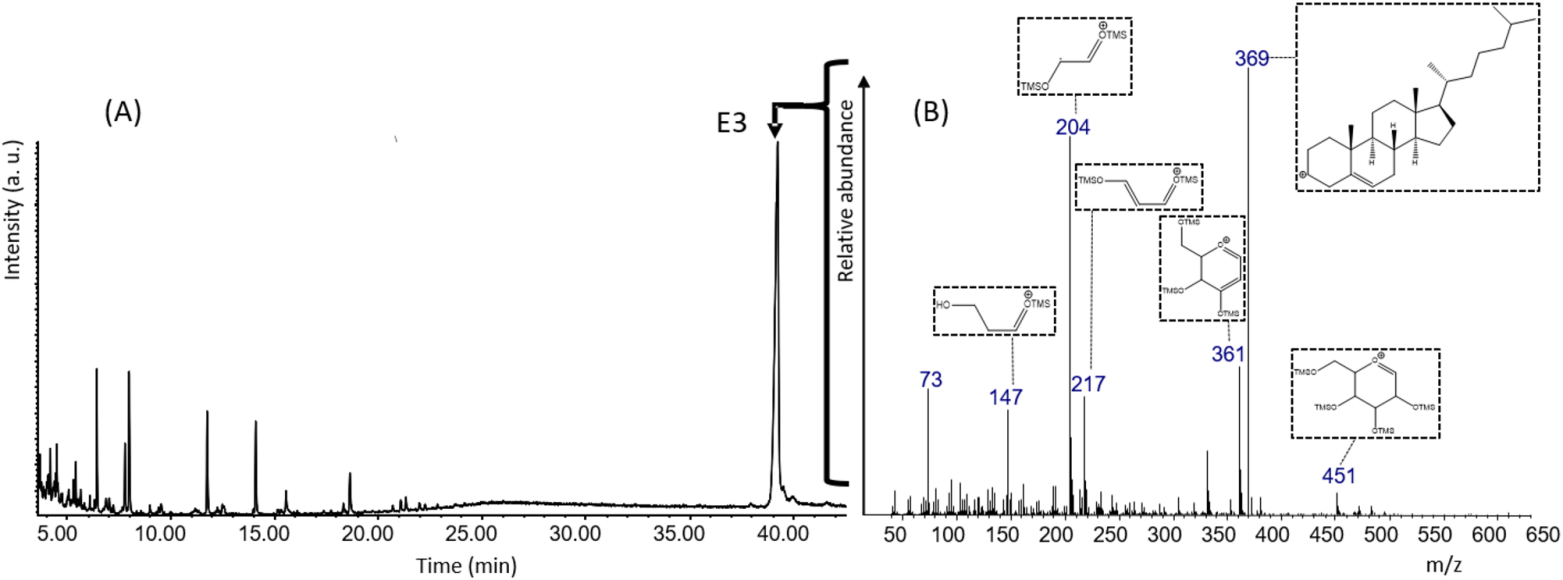
Gas chromatogram (**A**) of the fraction isolated through column chromatography and SPE, enriched in E3 (marked and highlighted with arrow) isolated from *H. suspectum* molt. MS spectra (**B**) of compound E3 tentatively identified as cholesteryl β-glucoside. The structure of each molecular ion is illustrated.

The total amount of extractable lipids in the air dry (H_2_O < 2 wt.%) *H. suspectum* molt is 9.7 ± 2.0 wt.% (the uncertain is reported hereafter as standard deviation). The GC–MS analysis shows a composition, which is qualitatively comparable with that obtained previously for other snakes^13^. This shows the presence of free fatty acids (35 ± 10 wt.%), free sterols (46 ± 15 wt.%, mostly cholesterol) and a significant higher contribution of a late eluting compound, E3, which is tentatively identified as a cholesterol derivative (Fig. 3). While the other lipids are similar to those of previous analyses^13^, it appears that the E3 component is a relevant constituent of the entire lipid fraction, with a content equal to 12 ± 4 wt.% of the lipid extract. Targeted analysis (with standards) for known ceramides (N-palmitoyl-D-erythro-sphingosine and N-stearoyl-D-erythro-sphingosine) related to xeric adaptation were performed. The GC–MS analysis of the *H. suspectum* epidermal molt lipid extract did not show any signal related to these ceramides (see experimental procedure).

**Figure 4:**
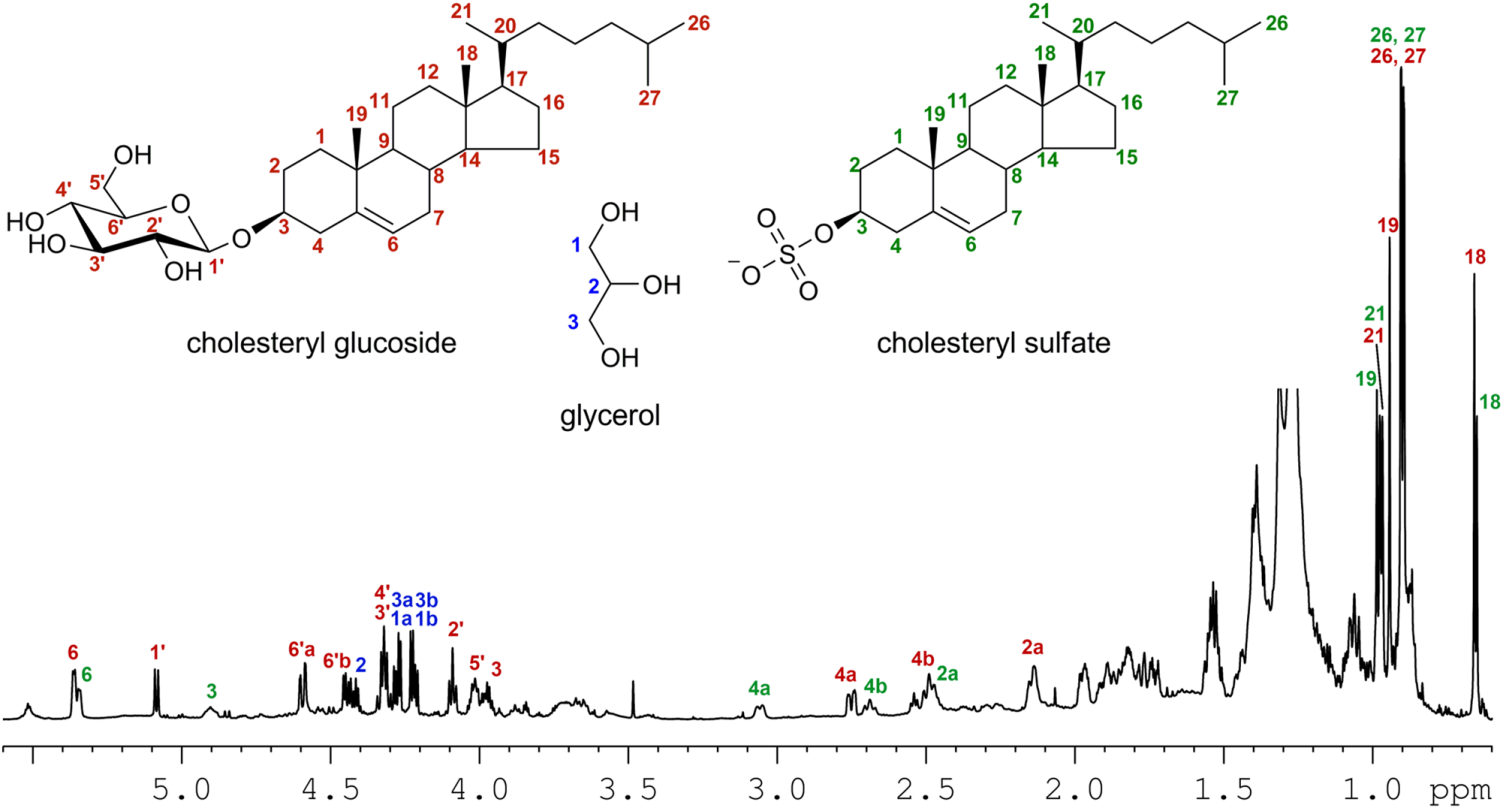
The ^1^H NMR spectrum of fraction E3 measured at 700 MHz using pyridine-d5 as solvent. This spectrum, together with the ^1^H NMR spectrum of the same fraction recorded in CD_3_OD (Fig. S5), allowed the identification of the three main components of the fraction. Signals that were assigned to the respective protons in the three compounds are marked with colored numbers. Those from cholesteryl β-glucoside are marked with red numbers, signals of cholesteryl sulfate with green numbers, and signals of glycerol with blue numbers. Side chain signals are coincident for the two sterols and are marked with black numbers. Signals of cholesteryl β-glucoside fully matched those reported in Maslov et al., 2010; signals of glycerol fully matched those of the spectrum of an authentic sample of glycerol in pyridine-d5 (Fig. S6). Cholesteryl sulfate was identified using the spectrum recorded in CD_3_OD (Fig. S5).

**Figure 5:**
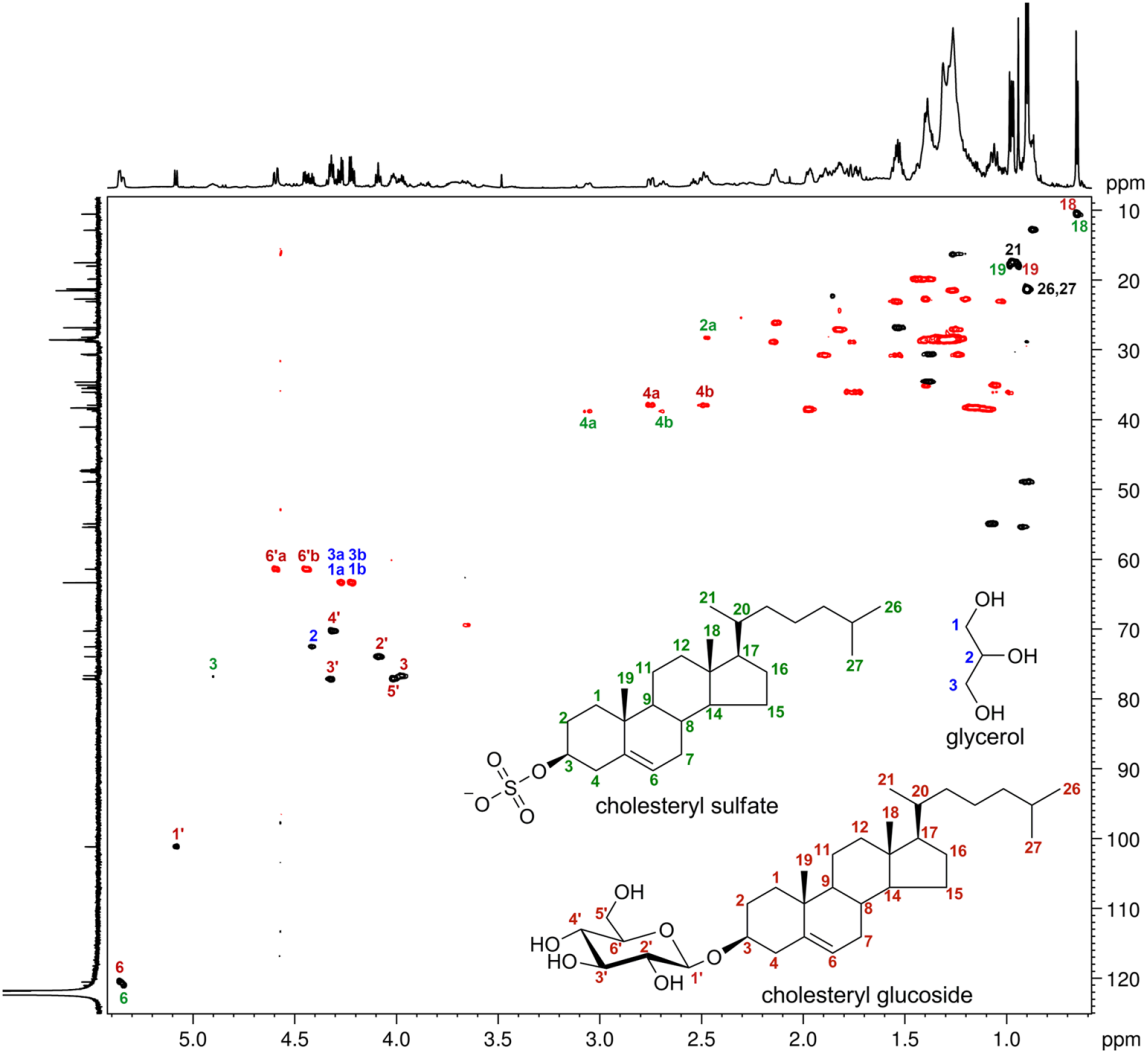
HSQC spectrum of fraction E3 recorded at 700 MHz using pyridine-*d*_5_ as solvent. Black signals refer to CH_3_ and CH groups, red signals refer to CH_2_ groups. Signals assigned to cholesteryl β-glucoside, cholesteryl sulfate, and glycerol are marked with red numbers, green numbers, and blue numbers, respectively. Side chain signals are coincident for the two sterols and are marked with black numbers.

The E3 fraction has been further investigated to clarify its specific composition. This was done by means of high-resolution LC–MS (Fig. S4) and NMR spectroscopy. ^1^H NMR spectra of the E3 fraction were recorded in pyridine-d5 (Fig. 4) and CD_3_OD (Fig. S5). They showed that fraction E3 is composed of cholesteryl β-glucoside [(3β)-cholest-5-en-3-yl β-D-glucopyranoside] (1.0), cholesteryl sulfate [(3β)-cholest-5-en-3-yl sulfate] (0.68) and glycerol (0.75) by comparison with NMR data reported in the literature (cholesteryl β-glucoside: Maslov et al., 2010; cholesteryl sulfate: Xiong et al. 2007)^21, 22^. The numbers in parentheses indicate the approximate molar ratios from integration of the ^1^H NMR spectrum recorded in pyridine-d5 (Fig. 4). Signals used for quantitation were δ 2.75 (1H, H-4a) for cholesteryl β-glucoside, 3.06 (1H, H-4a) for cholesteryl sulfate, and 4.21 (2H, H-1b and H-3b) for glycerol. This assignment was confirmed by high-resolution LC–MS data (Fig. S4) and was fully consistent with the HSQC, COSY and HMBC 2D NMR spectra (Figs. 5, Fig. S7 and S8).

## Discussion

### H. suspectum epidermal molt sampling

Here a study on the composition and structure of the epidermal molts from two individuals of Gila monster is reported. This epidermal molt has a unique lipid layer thickness (i.e. the mesos layer) that is much thicker than that observed in other species. The observations performed on the layers of this epidermal molts were highly reproducible and in agreement with what is reported in the literature^18, 19^. Thus, we are confident that the characterization performed is representative of the real composition and structure of the epidermal molts of the *H. suspectum*, even if only two individuals were used.

### Structural considerations

The SEM and TEM study confirms that the outermost, Oberhautchen, surface of *H. suspectum* is relatively smooth while, as reported in literature, mature mesos-cells are lenticular corneous scales loosely connected by an extracellular lipid material that is easily extractable (Fig. 1G)^2, 4, 8, 18, 23^. It is known that numerous bacteria, including *Helyobacter pylori*, contain cholesteryl beta-glucoside^24^, but bacteria were almost absent in our preparations since they are generally limited to the most superficial epidermal layers of reptilian epidermis^25^. Therefore, we confidently believe that the two cholesterol compounds here detected genuinely derive from the numerous corneocytes of the mesos-region of the analyzed molts. Moreover, the amount of these cholesterol compounds would be prohibitive to be all derived from bacterial contamination, being about 1 wt.% of the molt.

The investigation on the structure and composition of the molt shows that in *H. suspectum* are present many of the features observed in four other snake species previously investigated^13^. However, the relative thickness of the mesos-layer in relation to the beta-layer is much higher in *H. suspectum* than in the four snake species previously investigated as well as for most squamate species so far examined^1, 3, 4, 7–10, 23^. In fact, in the four species of snakes adapted to different ecological environments, from humid to more xeric conditions, the thickness of mesos-region was always less than the thickness of the beta-layer, at least by 25%. As opposed, in *H. suspectum* the mesos-region can be even thicker than the beta layer (Fig. 1B)^18^. The presence of these two layers also appears in the ATR-FTIR map that shows that the mesos-region has a composition very different from the beta-layer and is richer in methyl and methylene moieties, indicating the presence of hydrophobic moieties eventually from lipids. The ATR-FTIR technique also shows that the structural component of the corneous material of the mesos-region is enriched of about 10% in the disordered component at expense of the α-helix structure. The increase of disordered components in the corneous proteins present in mesos-cells reflects the much lower amount of proteins present in this region of the molt, in particular those with α-conformation with respect to molecules with random-coiled conformation^12, 13^. In fact, the mesos region is a transitional stratum between the hard, external beta-layer and the inner alpha-layer, where the relative amount of random coil component increases^3, 5, 6^.

### Lipid composition and epidermal barrier

Although possessing a thick mesos-region, the air dry molt from *H. suspectum* has a content of extractable lipids (about 10 wt.%) that is only slightly higher than those present in the snake species previously studied^13^. Similarly to what observed for snakes molts, the lipid extract shows an high content of free fatty acids (about 35% w/w_lipid_ or 3.5% w/w_molt_) and free sterols (about 46 w/w_lipid_ or 4.6% w/w_molt,_ mostly cholesterol) with a minor contribution of free ceramides. However, differently from snake molts of Rattlesnake, Tiger snake, Gaboon viper and Grass snake, the lipid extract from *H. suspectum* contains about 12% w/w_lipid_ of a peculiar lipid fraction, here indicated as E3 (Fig. 3). The E3 fraction was also detected (same retention time and MS spectra) in molts of rattlesnake and tiger snake, but was almost un-detected in these two species due to minor amount and, probably, a low response factor^13^. The results of the chemical characterization show that E3 fraction in *H. suspectum* is largely composed of cholesteryl β-glucoside (58 wt.%), cholesteryl sulfate (35 wt.%) and glycerol (7 wt.%). Therefore, the content of these lipids in the dry whole epidermal molts (w/w_molt_) was of 0.7 wt. %, 0.4 wt.% and 0.08 wt. %, respectively.

The co-presence of cholesteryl β-glucoside and cholesteryl sulfate is likely related to the stabilization of the mesos-layer and the heat shock response^26^. In fact, cholesteryl-beta-glucosyde is synthesized by a transfer reaction between glucosyl-ceramides and membrane cholesterol, often induced by heat shock. This reaction is localized in regions of the plasma membranes known as “lipid rafts”, and may be sensed by cellular mechanisms leading to a physiological response to the thermal damage due to exposure to high temperatures. One of the biochemical late responses to the initial heat shock is the formation of thermostable membrane domains^26^. In various studies it is reported that glycosylated steroids contribute to the stabilization of extracellular lamellar lipids and their attachment to cell membranes^1, 7, 9, 10^. The high incidence of this compound in *H. suspectum* epidermis further supports the idea that it derives from the large mass of mesos-cells observed in the molt (Fig. 1B,G). This glycolipid may play a role in the epidermal barrier function in snakes and it was proposed that it represents a biochemical vestige of a more primitive barrier-forming mechanism in reptiles^11^. Since this molecule represents an important lipid mediator of heat stress^26, 27^, its presence in relatively high amount in the epidermis of *H. suspectum* may also reflect the adaptation to the high temperature met by this lizard^15, 17^.

The sulfate moiety of the other cholesterol derivative, cholesterol sulfate, has a *pK*_a_ of ∼3.3, thus under physiologic conditions this molecule is ionized^28^. Thus, the sulfonation converts cholesterol, a rather rigid hydrophobic molecule, into an amphiphilic compound with a charged functional group. The hydrophobic/hydrophilic property of cholesterol sulfate is thought to make it ideally suited for interactions with membrane constituents, affecting its chemical-physical properties^28^. This molecule has been reported to be present in small amounts in chicken epidermis^11^ and in the human stratum corneum and plasma^29^.

Both these two molecules represent soluble forms of cholesterol that can be transported in other regions of the membrane or in the cytoplasm of epithelial cells. The presence of these two steroid compounds suggests that they may be indicators of cellular mechanisms commonly present in the epidermis of this heat-adapted species to sun exposition that somehow sense the effect of heat on the membrane of keratinocytes of the epidermis. It can be speculated that together other stimuli the heat damage eventually influences retreat of the lizard into shadowed areas to reduce the damage derived from the thermal exposure. Since *H. suspectum* possess an EWL value even higher than in other lizards adapted to temperate or semi-desert conditions^14^, the role of the very thick mesos-layer detected in this species is essential to limit water loss^18^. In fact, since most of total water-loss derives from cloacal evaporation, a physiological mechanism utilized in this species for body cooling, the thick mesos layer present in the epidermis of most body regions contributes to the adaptation of this lizard to the high environmental temperatures of the Sonoran Desert^14, 17^. The increase of circulating fluid osmolarity during active life under extreme heat and sun-light conditions is counterbalanced from the reduction of the rate of dehydration derived from the water reservoir present in the urinary bladder of this species^16^. The present study suggests that, in addition to the other physiological and behavioral adaptations, this signaling mechanism may represent an essential component for survival, together with other behavior adaptations present in this species against sun exposition^19^ and water conservation^14, 16, 17^.

In summary, the epidermis of this species was studied to analyze the chemical composition of the lipids since it presents a mesos layer much thicker than the mesos layer observed in other species. Vibrational spectroscopy and electron microscopy data revealed that the mesos layer is richer in lipids as compared to the other layers of the molt. In particular, the chromatographic profile of the extracted lipids revealed a relative fraction (E3, about 10 wt.%) than is much higher in *H. suspectum* in comparison to other investigated species. Of relevance, the analysis of this E3 fraction showed the presence of two lipids, so far not reported in other snake and lizard molts, which can have implications in structuring the membrane of the mesos layer’s cells and in controlling water trafficking. The importance of these lipids for the ecology of the species remains to be specifically analyzed.

## Materials and methods

### Tissue collection and preparation

The shed molts from two individuals of Gila monster (*Heloderma suspectum*) kept in a terrarium under similar conditions of the Arizona desert, were kindly donated from Dr. Bob Meyer (Rattlesnake Museum, Albuquerque, NM, USA). These tissues were used for the experiment. Other tissues were re-studied from two *H. suspectum* molts previously prepared and embedded in resin^18^ They were used for transmission electron microscopy observations. Briefly, tissues were fixed for 5–8 h in 2.5% glutaraldehyde in 0.1 M phosphate buffer at pH 7.4, post-fixed in 2% OsO_4_, dehydrated in ethanol, and finally embedded in Durcupan Resin.

### Transmission electron microscopy observations

Transmission electron microscopy (TEM) observations were performed on thin sections with a thickness in the range 50–80 nm. These sections were later collected on copper grids of 200–300 mesh, counterstained with uranyl acetate and lead citrate according to routine procedures, and then studied under a Zeiss C/10 transmission electron microscope operating at 60 kV. Images were collected using a digital camera and imported into a computer operating with a Photoshop 8.0 program.

### Scanning electron microscopy observations

SEM observations were performed on unstained molts of *H. suspectum*. Both surfaces and cross-section of the molt were investigated. The cross section was prepared mechanically breaking a molt fragment frozen in liquid nitrogen. The molt specimens were coated with a 2 nm thick layer of gold using a gold sputter before the observations. The specimens were studied using a FEG Hitachi 6400 scanning electron microscope operating at 15 kV.

### Attenuated Total Reflectance Infrared Spectroscopy (ATR-FTIR) analysis

ATR-FTIR spectra were acquired in the range 4000–650 cm^−1^ with a Thermo-Nicolet Nexus 5700 spectrometer connected to a Thermo Continuum IR microscope fitted with an MCT type A detector cooled by liquid nitrogen as reported in Torri et al.^13^. Briefly the sample cross-section was prepared as follows. A piece of molt was pressed (10 tons) between KBr layers obtaining a disk. Successively, the disk was polished edge-on so that a sample cross-section was exposed. For the measurements a 15 × Thermo-Electron Infinity Reflachromat objective was used and a tube factor of 10 × was used in reflection mode. The 15 × objective was connected to a micro slide-on ATR with a silicon crystal. The contact area diameter was of 120 μm. The ATR mapping of selected areas of the sample was carried out. The data were analyzed using the Nicolet ‘‘Omnic-Atlls’’ software (Thermo Electron Corporation, Madison, WI). The set of spectra were collectively treated. This means that instead to correct the background on the single spectrum the entire data set were baseline-corrected.

The curve fitting to Gaussian line shapes over the 2000–900 cm^−1^ region (Fig. S2 and S3) was performed using the Nicolet ‘‘Omnic-Atlls’’ software (Thermo Electron Corporation, Madison, WI). The amide I region was curve-fit into the individual component absorption peaks and these spectra were used to identify the component absorptions attributed to α-helix, β-sheets and disordered microstructural components of the epidermal snake molt. The amount of these structural components was determined as percentage by adding the sum of absorptions for each band and expressing their sums as a fraction of the total amide I. Amide II region and the region 2800–3000 cm^−1^ were also analyzed in their band components, similarly to Amide I region.

### Chemical analysis

In a typical experiment on 190 mg of *H. suspectum* molt, a solvent extraction was applied. As solvent, a refluxing dichloromethane:methanol mixture in 2:1 volume ratio was utilized for 24 h at 60 °C. The extraction residue (defatted exuviae) was removed by settling and the resulting extract was evaporated, yielding 18.5 mg of solid lipid. This procedure was repeated twice on molt from the same organism and the same yield of lipids was obtained. The limited availability of molt did not allow further lipid extraction experiments.

Gas chromatography-mass spectroscopy (GC–MS) analysis was performed directly on 1 mg of the extract, which was subjected to silylation ad GC–MS analysis with high temperature thermal program, following previously published procedure^13^. The GC–MS analysis showed presence of free fatty acids, free sterols (mostly cholesterol), and a significant higher contribution of a late eluting compound (E3). Calibration was performed using Ergosterol as Internal Standard, assuming unitary response factor, which provided a quantitative estimate of E3.

To isolate the E3 and to proceed with its chemical identification, the extract was subjected to silica gel chromatography with sequential elution with ciclohexane, ethylacetate:cyclohexane (2:8,5:5,8:2,10:0) followed by methanol. The fraction eluted with methanol resulted enriched in the target compounds, without contamination by free sterols, but with still significant amounts of free fatty acids. This fraction was further purified by flash SPE (6 ml, 1 g DSC-Si Supelco washed with ethyl acetate, dried and conditioned with 5 ml n-hexane), performed with sequential elution using 5 ml of dichloromethane and 5 ml of dichloromethane:methanol (1:1). The latter fraction provided about 0.5 mg of the isolated compound that was identified as E3 with 85% GC–MS purity.

### Ceramide analysis

As standard ceramides the N-palmitoyl-D-erythro-sphingosine (Merck) and the N-stearoyl-D-erythro-sphingosine (Merck) were used. Aliquots of ceramide standard solutions were prepared. They were subjected to the same silylation-GC–MS procedure used for analysis of epidermal molt extract. This procedure was performed using a ceramide amount equal to that expected in a lipid extract with 5 wt. % (on lipid extract) ceramide. GC–MS analysis of the standard ceramides revealed clear and sharp peaks in the late part of chromatogram. However, we are unable to detect those peaks in the lipid extract chromatogram. Thus, we can assume safely that ceramide content of extract should be significantly less than 5 wt. %.

### Analysis of E3 from H. suspectum

Composition of the E3 fraction was investigated by high-resolution Liquid Chromatography-Mass Spectroscopy (LC–MS) using a Thermo LTQ-Orbitrap XL system, and by 1D (^1^H and ^13^C) and 2D (COSY, HSQC and HMBC) NMR using a Varian UnityInova spectrometer at 700 MHz equipped with a cryogenic probe. NMR chemical shifts were referenced to the residual solvent signal (CD_3_OD: δ_C_ 49.0, δ_H_ 3.31; pyridine-*d*_5_ δ_C_ 150.3, 135.9 and 123.9, δ_H_ 8.73, 7.56 and 7.21).

## Supplementary information

Supplementary file1

## Data Availability

The following files are available free of charge in a single Word document: detailed ATR-FTIR data, LC–MS analysis, and mono- and bi-dimensional NMR spectra.
