# Supplementary material for: Cholesterol derivatives make large part of the lipids from epidermal molts of the desert-adapted Gila monster lizard (Heloderma suspectum)
Source: Sci Rep. 2020 Oct 14;10:17197. doi: 10.1038/s41598-020-74231-5 (PMC7566651; doi:10.1038/s41598-020-74231-5)
Supplement: Supplementary file 1 — Supplementary file1 [file 41598_2020_74231_MOESM1_ESM.docx]

**Supporting Information**

Cholesterol derivatives make large part of the lipids from epidermal molts of the desert-adapted Gila monster lizard (*Heloderma suspectum*)

Cristian Torri^$^, Giuseppe Falini^+;*^, Devis Montroni^+^, Simona Fermani^+^, Roberta Teta^&^, Alfonso Mangoni^&^, Lorenzo Alibardi^#, *^

^+^ Department of Chemistry “Giacomo Ciamician”, University of Bologna, via Selmi 2, 40126, Bologna, Italy

^&^ Department of Pharmacy, University of Napoli Federico II, via Montesano 49, 80131 Napoli, Italy

^$^ Department of Chemistry “Giacomo Ciamician”, University of Bologna Campus of Ravenna, via S. Alberto 163, Ravenna, Italy

^#^ Comparative Histolab Padova, Italy

Table S1 pag. S2

Table S2 S3

Figure S1 S4

Figure S2 S5

Figure S3 S6

Figure S4 S7

Figure S5 S8

Figure S6 S9

Figure S7 S10

Figure S8 S11

**Table S1**. ATR-FTIR absorption bands (cm^-1^) of *H. suspectum* epidermal molt.

| β | m | m-α |  | Assignments |
| --- | --- | --- | --- | --- |
| 3282s | 3262s | 3282s |  | ν_OH_ |
| 3070w | 3020w | 3020w |  | ν_CH_ |
| 2962w  2926m | 2955m  2919s | 2955m  2919s |  | ν_CH2_ |
| 2875w  2853w | 2875w  2851s | 2875w  2851s |  | ν_CH3_  ν_CH2_ |
| 1644s | 1644s | 1644s |  | ν_CO_, amide I |
| 1539s  1512s | 1539s  1512m | 1539s  1512w |  | ν_CO_-δ_NH_, amide II |
| 1451m | 1451m | 1451m |  | δ_CH2_ |
| ~1410w | ~1400w | ~1400w |  | δ_CH3_ |
| 1239m | 1239m | 1239m |  | ν_CN_, amide III |
| 1030w | 1030m | 1030s |  | ν_COC_ |

The subscript indicates the relative intensity of the diffraction bands. s indicates strong. m: indicates medium. w indicates weak.

**Table S2**. Data from the analyses of the different components of the FTIR spectra as illustrated in figure S2 and S3.

|  | beta-layer | |  | beta/meso-layer | |  | meso-layer | |
| --- | --- | --- | --- | --- | --- | --- | --- | --- |
|  | band  (cm^-1^) | rel. intensity  (%) |  | band  (cm^-1^) | rel. intensity  (%) |  | band  (cm^-1^) | rel. intensity  (%) |
| Amide II | 1513.5 | 42.95 |  | 1511.6 | 35.52 |  | 1511.6 | 35.37 |
|  | 1539.0 | 30.22 |  | 1539.0 | 31.41 |  | 1539.1 | 30.69 |
|  | 1567.5 | 26.83 |  | 1559.2 | 33.07 |  | 1568.7 | 33.94 |
| Amide I | 1621.7 | 37.34 |  | 1624.3 | 37.50 |  | 1620.8 | 36.14 |
|  | 1646.1 | 42.80 |  | 1650.3 | 42.24 |  | 1644.2 | 31.13 |
|  | 1678.3 | 19.86 |  | 1678.5 | 20.26 |  | 1674.3 | 32.74 |
| 2800-3000 cm^-1^ |  | 13.3* |  |  | 19.3* |  |  | 19.8* |

* Relative integrate intensity of the absorption bands in the region 2800-3000 cm^-1^ with respect to the amide I and amide II absorption bands.


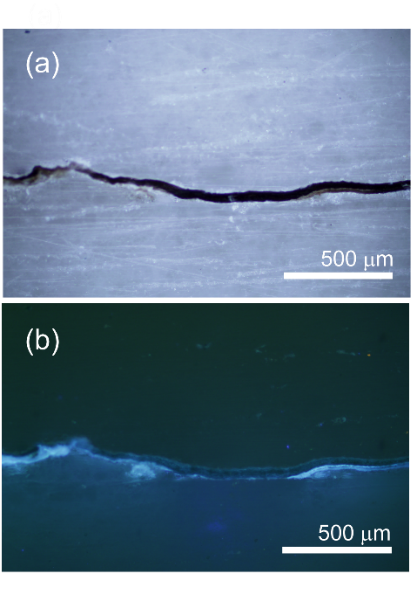


**Figure S1**. (a) Camera picture of the cross-section of molt from *H. suspectum* and the corresponding (b) fluorescence image.


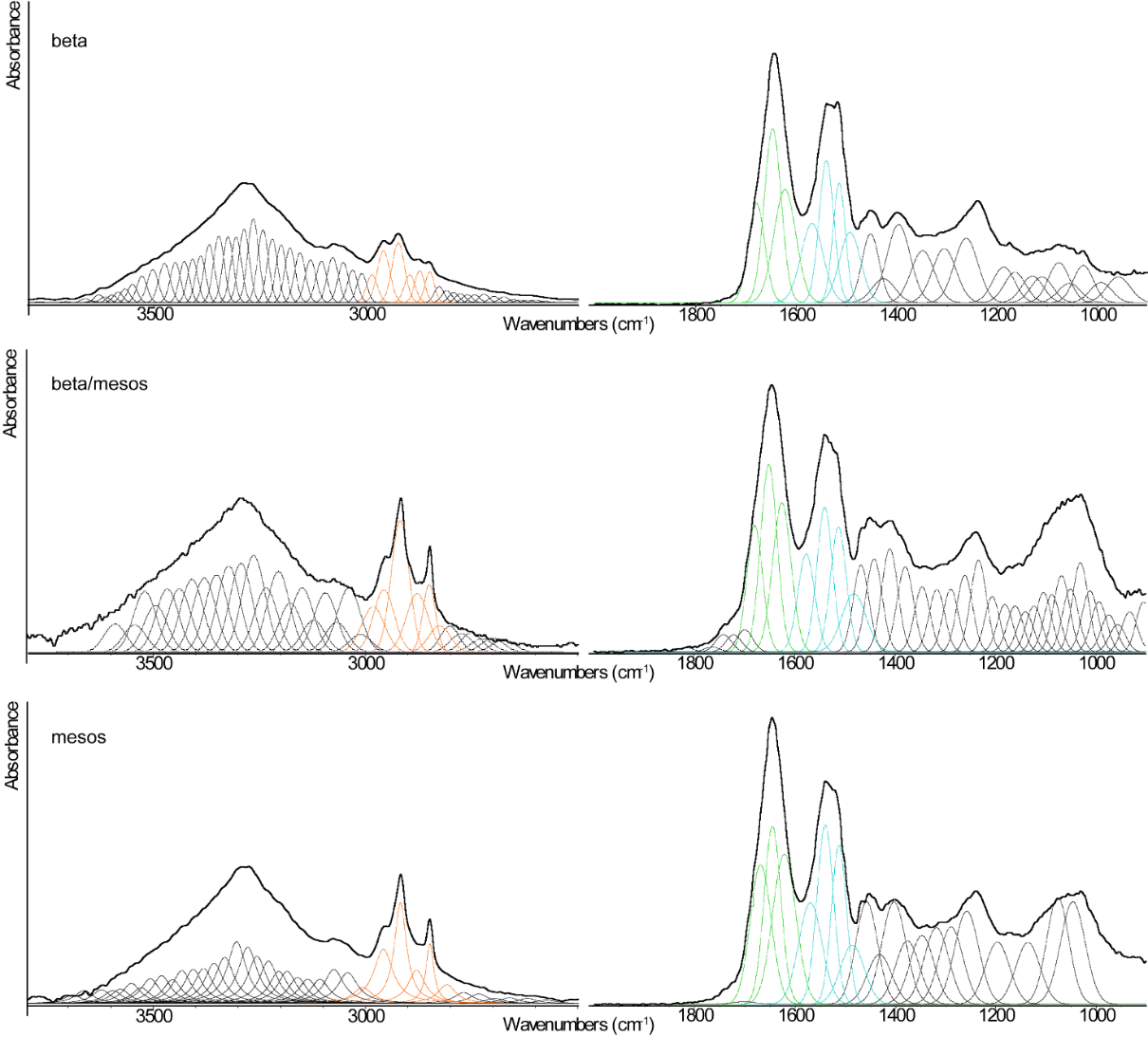


**Figure S2**. Deconvolution of the main absorption bands of ATR-FTIR spectra from the three different regions in the molt cross-section of *H. suspectum*, which are beta (top), beta/mesos (middle) and mesos (bottom). The absorption bands with de-convoluted in green, blue and orange correspond to the amide I, amide II and methyl-methylenic vibration modes, respectively.


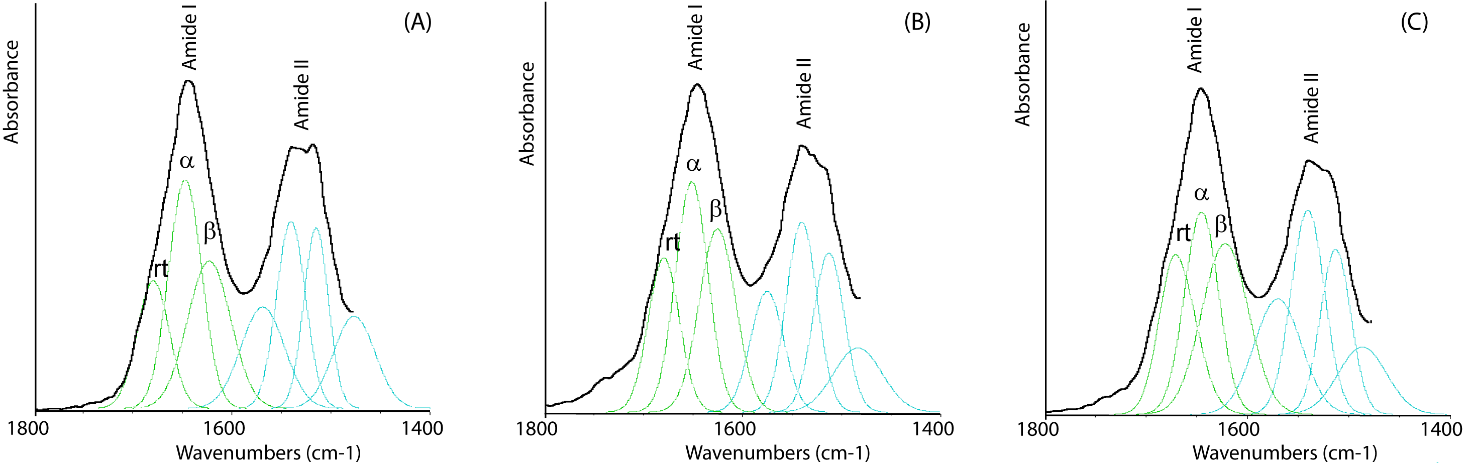


**Figure S3**. Deconvolution of the amide I and amide II absorption bands of ATR-FTIR spectra from the three different regions in the molt cross-section of *H. suspectum*, which are beta (left), beta/mesos (middle) and mesos (right). The labels α, β, and rt indicate the amide I structural components, α-helix, β-sheet and turn, loop and random coil, respectively, considered for the fitting.


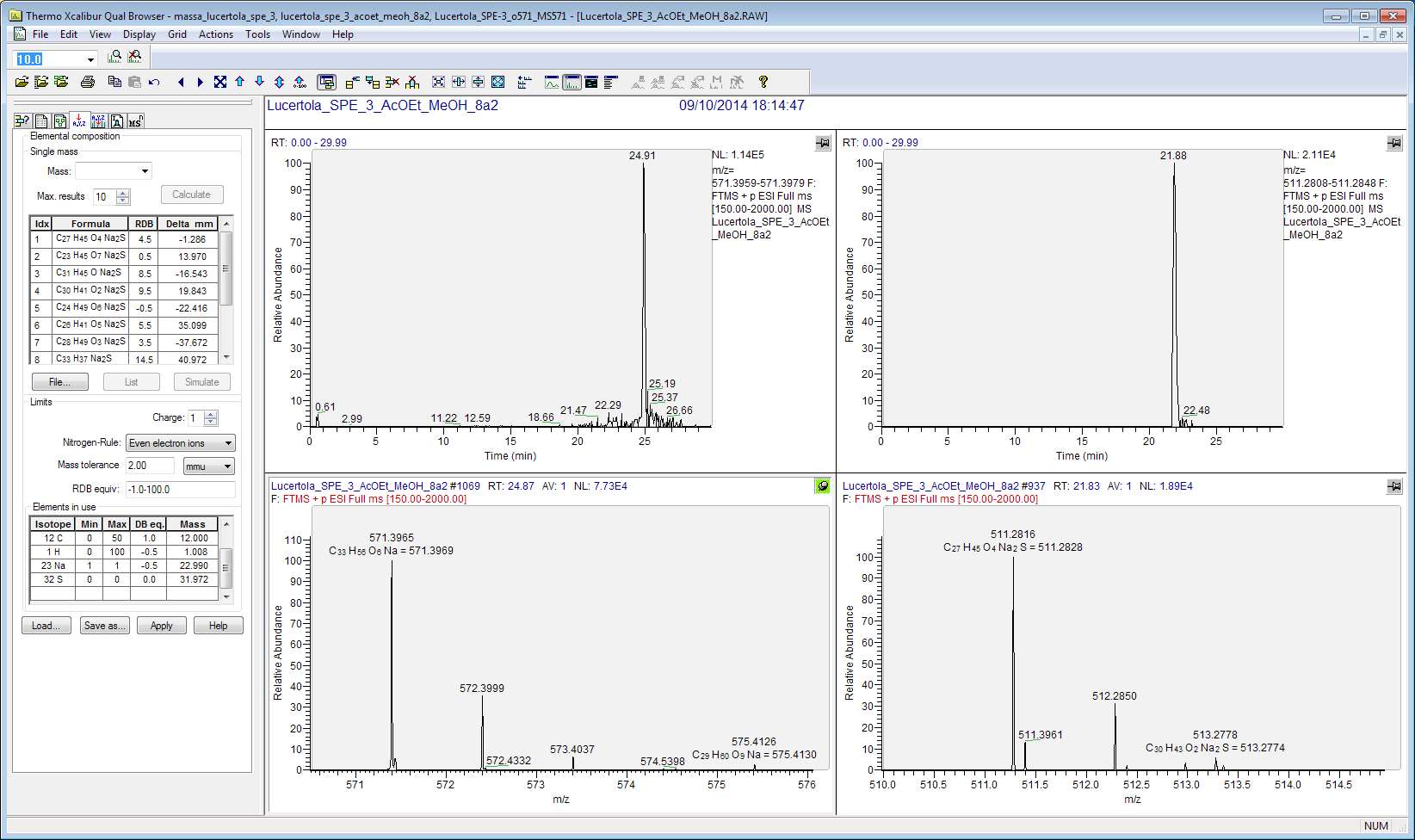


**Figure S4**. The extracted ion chromatograms from the LC-MS analysis of fraction E3 at *m/z* 571.3969 (corresponding to the [M+Na]^+^ ion from cholesteryl β-glucoside,) and at *m/z* 511.2828 (corresponding to the [M–H+2Na]^+^ ion from cholesteryl sulfate), confirming the .presence of these compounds in the fraction.


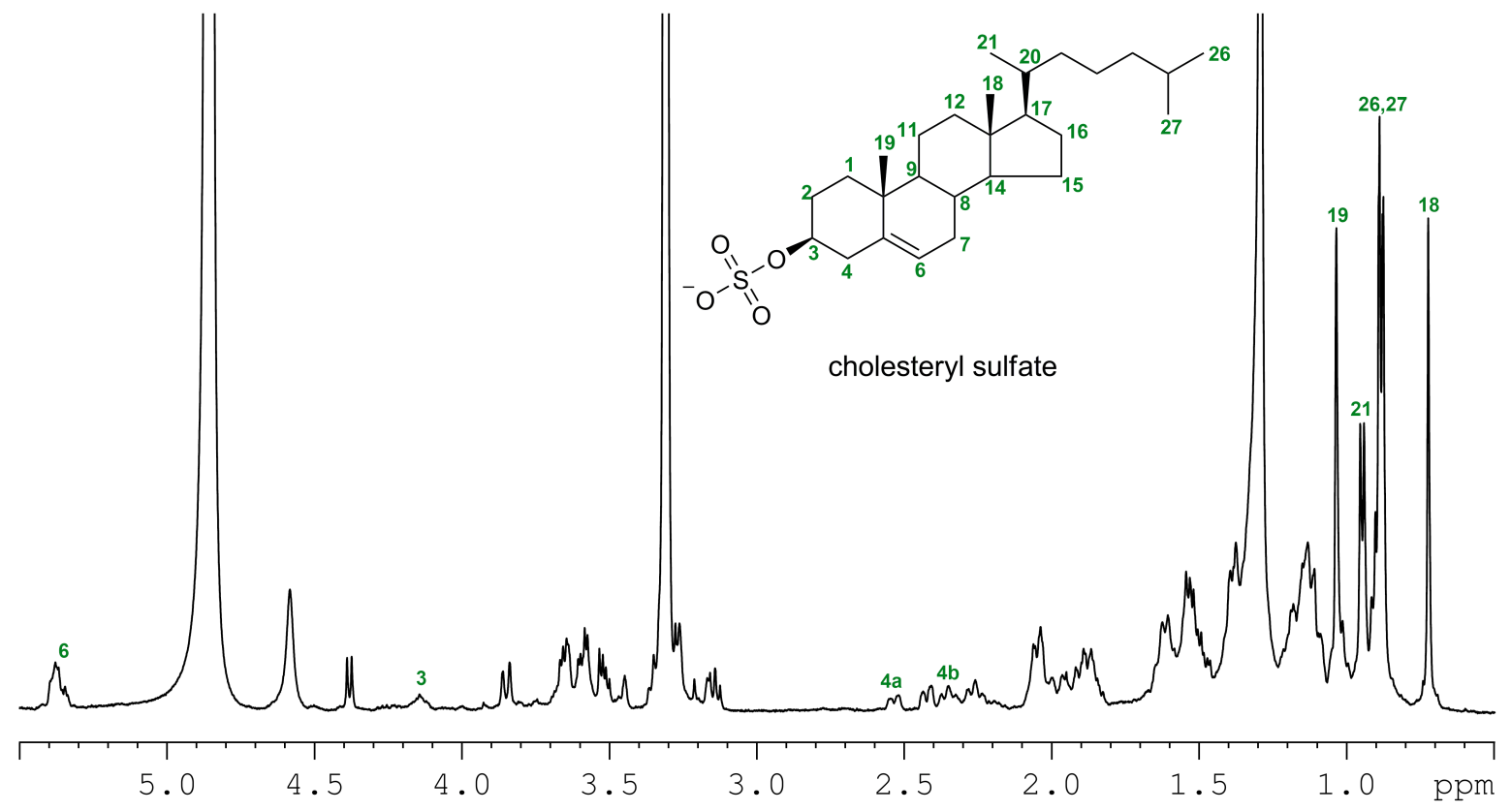


**Figure S5**. ^1^H NMR spectrum of fraction E3 recorded at 500 MHz using CD_3_OD as solvent. Assigned signals of cholesteryl sulfate (green numbers) fully matched those reported in Xiong et al., 2007 (in particular, the de-shielded proton of H-3 at δ 4.13).


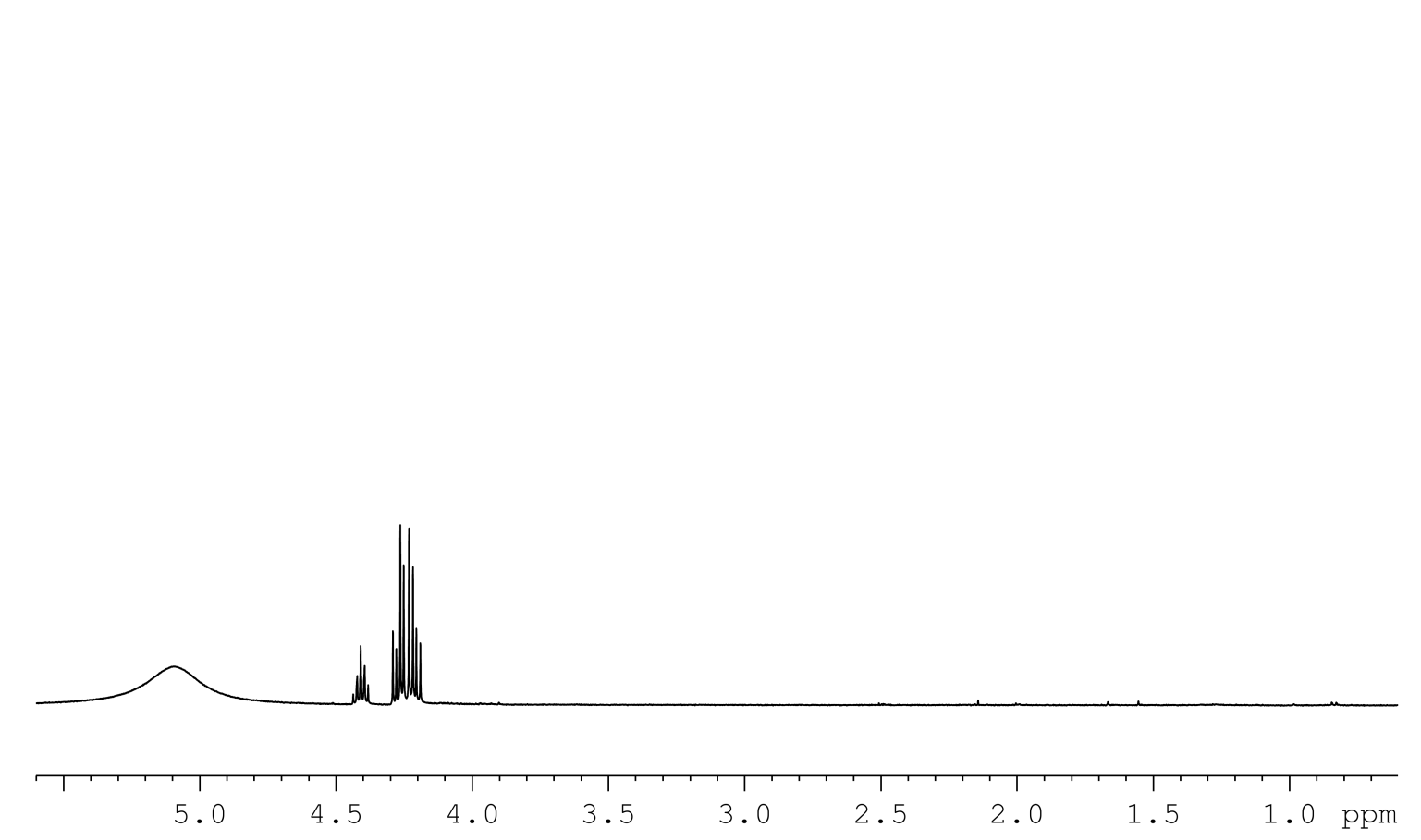


**Figure S6**. ^1^H NMR spectrum of an authentic sample of glycerol recorded at 400 MHz using pyridine-d5 as solvent. NMR parameters: δ 4.41 (apparent quintet, *J* = 5.5 Hz), 4.27 (dd, *J*= 10.8 and 5.0 Hz), 4.21 (dd, *J*= 10.8 and 5.8 Hz).


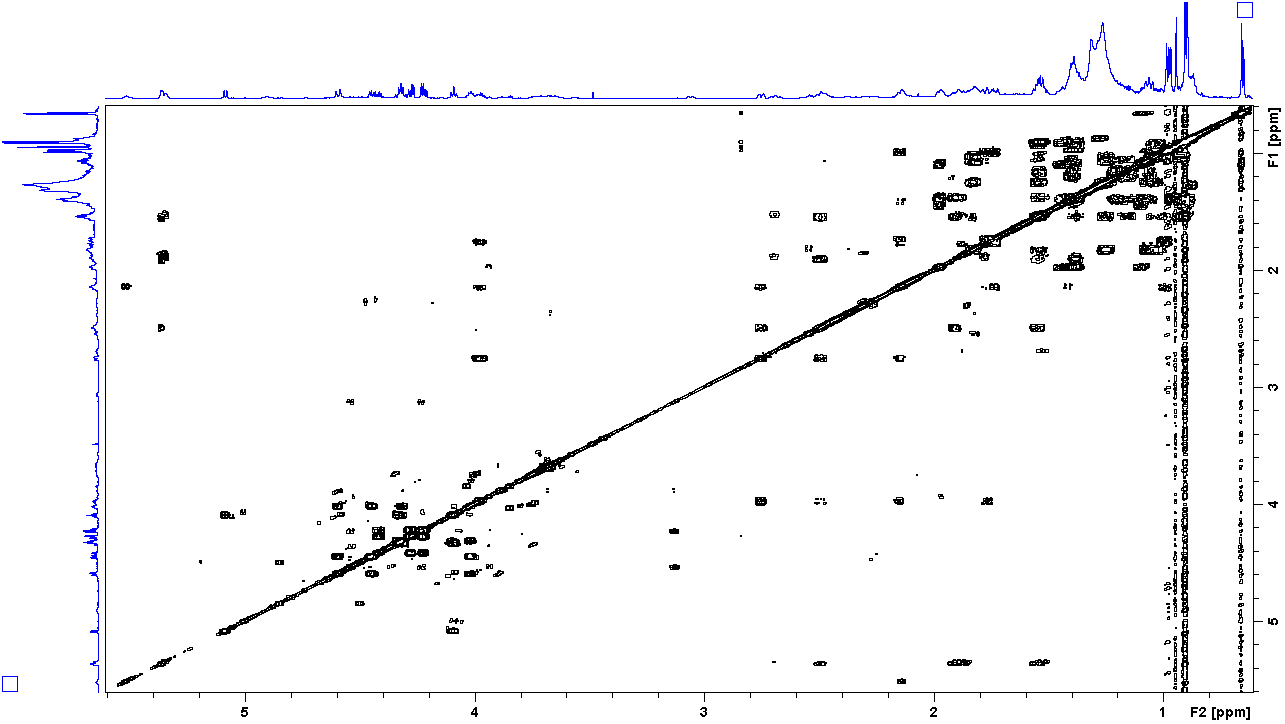


**Figure S7**. COSY spectrum of fraction E3 recorded at 700 MHz using pyridine-*d*_5_ as solvent.


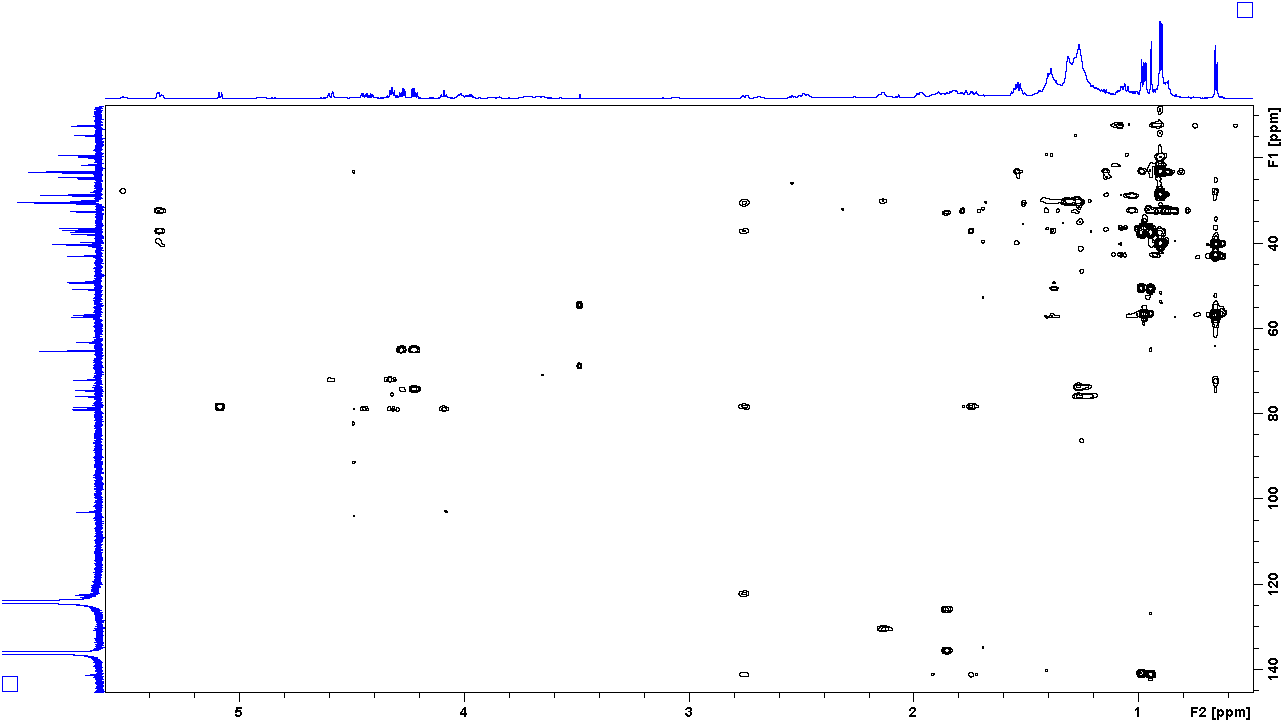


**Figure S8**. HMBC spectrum of fraction E3 recorded at 700 MHz using pyridine-*d*_5_ as solvent.
